# 

**Published:** 1801-10

**Authors:** 


### To CORRESPONDENTS.

*Mr. Paget's drawings are put into the hands of an eminent artist, and are promised for our next Number.*

*The Medical Intelligence from Manchester came too late for insertion in our last; and our Correspondent will perceive, by the contents of the present Number, that it is no longer* News.

*Communications are received from Messrs.* Moffat, Blair, *and* Reece.

---

### ERRATA.

Vol. VI. p. 197, l. 5, *for* ℨj. muriatic acid, *read* ʒj.
p. 234, l. 7, *dele* septem punctata.

---

### ADVERTISEMENT.

*Persons who reside abroad, and who wish to be supplied with this Work every month as published, may have it sent to them,* FREE OF POSTAGE, *to New York, Halifax, Quebec, and every part of the West Indies, at three guineas per annum, by Mr.* Thornhill, *of the General Post Office, at No.* 21, *Sherborne Lane; to Hamburgh, Lisbon, Gibraltar, and every part of the Mediterranean, at three guineas per annum, by Mr.* Bishop, *of the General Post Office, at No.* 22, *Sherborne Lane; to the Cape of Good Hope, or to any part of the East Indies, at two guineas per annum, by Mr.* Guy, *of the India House; and to any part of Ireland, at two pounds ten shillings per annum, by Mr.* Smith, *of the General Post Office, at No.* 3, *in Sherborne Lane. It may also be had of all persons who deal in Books in every part of the world.*

